# Outcomes of re-treatment with first-line trastuzumab plus a taxane in HER2 positive metastatic breast cancer patients after (neo)adjuvant trastuzumab: A prospective multicenter study

**DOI:** 10.18632/oncotarget.9331

**Published:** 2016-05-27

**Authors:** Binghe Xu, Xichun Hu, Hong Zheng, Xiaojia Wang, Qingyuan Zhang, Shude Cui, Donggeng Liu, Ning Liao, Rongcheng Luo, Qiang Sun, Shiying Yu

**Affiliations:** ^1^ Department of Medical Oncology, Cancer Hospital, Chinese Academy of Medical Sciences, Peking Union Medical College, Panjiayuan, Chaoyang District, Beijing, China; ^2^ Department of Medical Oncology, Fudan University Shanghai Cancer Center, Department of Oncology, Shanghai Medical College, Fudan University, Shanghai, China; ^3^ Laboratory of Molecular Diagnosis of Cancer, State Key Laboratory of the Biotherapy and Cancer Center, West China Hospital, Sichuan University, Chengdu, China; ^4^ Department of Medical Oncology, the Key Laboratory of Integrated Chinese and Western Medical Oncology in Zhejiang Province, Zhejiang Cancer Hospital, Hangzhou, China; ^5^ Department of Medical Oncology, Harbin Medical University Cancer Hospital, Harbin, China; ^6^ Breast Disease Center, Henan Cancer Hospital & Affiliated Cancer Hospital, Zhengzhou University, Zhengzhou, China; ^7^ Department of Medical Oncology, State Key Laboratory of Oncology in South China, Sun Yat-sen University Cancer Center, Guangzhou, China; ^8^ Department of Breast Cancer, Cancer Center, Guangdong General Hospital, Guangzhou, China; ^9^ Cancer Center, Southern Medical University, Guangzhou, China; ^10^ Department of Breast Surgery, Peking Union Medical College Hospital, Beijing, China; ^11^ Cancer Center, Tongji Hospital of Tongji Medical College, Hua Zhong University of Science and Technology, Wuhan, China

**Keywords:** trastuzumab, human epidermal growth factor receptor 2 (HER2), metastatic breast cancer, (neo)adjuvant trastuzumab, re-treatment

## Abstract

Trastuzumab is the backbone of HER2-positive early breast cancer (eBC) and metastatic breast cancer (mBC) treatment, but limited data exist as to re-treatment in relapsed patients. In this prospective, single arm, multicenter trial, we assessed efficacy and safety of trastuzumab and taxane combination in Chinese patients with HER2-positive mBC relapsed after prior (neo)adjuvant trastuzumab. Patients with previous (neo)adjuvant trastuzumab treatment for≥9 weeks and a relapse-free interval ≥6 months were assigned to trastuzumab treatment with paclitaxel or docetaxel. The primary endpoint was progression free survival (PFS). Secondary endpoints included overall response rate (ORR), clinical benefit rate (CBR), duration of response (DOR), time to progression (TTP), overall survival (OS) and safety profile. Thirty-two patients were enrolled and treated for a median duration of 33.5 weeks. The median PFS was 9.9 months (95% CI, 6.28 - 13.63 months). The ORR was 81.3% (95% CI, 63.6% - 92.8%) and CBR (CR+PR+SD≥6months) was 81.3% (95% CI, 63.6% - 92.8%). The median DOR was 9.8 months (95% CI, 5.82 - 11.60 months) and median TTP was 9.9 months (95% CI, 6.28-13.63 months). OS median follow-up time was 20.1 months and 25% OS time was 25.5 months. The safety profile was acceptable with common adverse events including leukopenia (59.4%), neutropenia (56.3%), hypoaesthesia (34.4%) and granulocytopenia (31.3%). In conclusion, re-treatment with trastuzumab plus a taxane as first-line therapy is an effective regimen for patients with HER2-positive mBC relapsed after (neo)adjuvant trastuzumab. The safety profile was good and the adverse reactions were tolerable and manageable.

## INTRODUCTION

Breast cancer is the most common cancer in women among all racial and ethnic groups by a wide margin [1]. In China, the number of female breast cancer cases was estimated to be 248,620 in 2014, with a crude incidence rate of 37.86 per 100,000 [2]. Around 20-30% of breast cancer cases are characterized with an overexpression of the human epidermal growth factor receptor 2 (HER2), and HER2-positive breast cancer is associated with increased aggressiveness in disease progression and worse clinical outcomes including lower disease-free survival and overall survival (OS) [3, 4].

Trastuzumab has been used as the standard of care for HER2-positive early breast cancer (eBC) and metastatic breast cancer (mBC) [5, 6]. In (neo)adjuvant setting as reported in the NOAH trial, treatment with trastuzumab plus chemotherapy showed a 3-year event-free survival of 71% *versus* 56% compared with chemotherapy alone in patients with HER2-positive breast cancer [7]. However, relapse after (neo)adjuvant trastuzumab treatment for HER2-positive eBC still occurs at a significant rate [8, 9], and tumor cells may develop trastuzumab-resistance. In the previous pivotal combination trials (H0648g and M77001), trastuzumab plus a taxane as first-line treatment in HER2-positive mBC patients showed a significant clinical benefit compared to chemotherapy alone [10, 11]. More recently, several new anti-HER-2 agents such as pertuzumab, trastuzumab emtansine (T-DM1), and Lapatinib have been developed [12-16]. However, the eligible patients in most of the trials studying the above anti-cancer agents were trastuzumab-naïve, thus their clinical outcomes in patients who develop recurrent disease from (neo)adjuvant trastuzumab setting are still largely unknown. Increasing evidence reported the effectiveness of continuous blockade of HER2 by trastuzumab, including two retrospective studies which have shown the efficacy of re-treatment regimen with trastuzumab in HER2-positive breast cancer, reporting an OS of 48.2 months [17] and two-year OS rate of 60.0% [18]. Re-treatment after Herceptin Adjuvant Trial reported with a median progression free survival (PFS) of 8.0 months and overall survival of 25.0 months in HER2-positive mBC patients relapsed after adjuvant trastuzumab [19].

Thus, given the promising results but still limited data in the outcomes of re-treatment with trastuzumab, we performed a multicenter, single arm, open-label study to assess the efficacy and safety of first-line trastuzumab in combination with a taxane in patients with mBC who relapsed after receiving (neo)adjuvant trastuzumab for HER2-positive eBC in a Chinese population.

## RESULTS

### Baseline characteristics

This multicenter, open label, single arm study enrolled patients from February 10, 2011 through May 3, 2013. A total of 32 eligible patients from 11 study centers were enrolled, and the clinical cut-off date for analysis was July 14, 2014.

The baseline demographic data and characteristics of the enrolled 32 HER2-positive female patients (Intention to treat [ITT] population) are summarized in Table 1. Overall, the subjects had a median age of 48 years (25-74 yr). The Eastern Cooperative Oncology Group (ECOG) score during the screening period was 0 for 19 patients (59.4%) and 1 for 13 patients (40.6%). Four patients had abnormal baseline electrocardiogram (ECG) test (12.5%). The medical history of the patients showed a median time from the histological diagnosis of primary breast cancer to enrollment of 33.7 months ranging from 13.2 months to 114.3 months, with evenly distributed clinical stages at I (10.3%), IIA (24.1%), IIB (27.6%), IIIA (13.8%), IIIB (3.4%), and IIIC (20.7%). Twenty four patients (75.0%) had received the chemotherapy with anthracyclines in which 23 patients had received it as adjuvant chemotherapy and 5 patients had received it as neoadjuvant chemotherapy. The median withdrawal time from (neo)trastuzumab prior to this enrollment was 21.38 months ranging from 6.41 months to 95.89 months. The median number of cycles of prior trastuzumab treatment was 18 periods (ranging 3 - 63 periods). Furthermore, all the 32 patients had undergone prior surgeries, including lymphadenectomy and axillary surgery for all patients (100%), mastectomy for 26 patients (81.3%), lumpectomy for 8 patients (25.0%), and other surgeries (expander implantation, nodulectomy of the left chest wall) for 2 patients (6.3%).

**Table 1:** Demographic data and Baseline Characteristics (ITT)

Characteristics	*ITT (N=32)*
Median age (years), range	48, 25-74
ECOG performance status, n (%)	
0	19 (59.4%)
1	13 (40.6%)
Hormone receptor status, n (%)	
Estrogen receptor	
Positive	8 (25.0%)
Negative	24 (75.0%)
Progesterone receptor	
Positive	7 (21.9%)
Negative	25 (78.1%)
Previous therapy, n (%)	
Anthracycline	24 (75.0%)
Neoadjuvant	5 (15.6%)
Adjuvant	23 (71.9%)
Taxane	29 (90.6%)
Neoadjuvant	7 (21.9%)
Adjuvant	28 (87.5%)
Other agents	27 (84.4%)
Neoadjuvant	5 (15.6%)
Adjuvant	27 (84.4%)
Radiotherapy	18 (56.3%)
Neoadjuvant	0 (0.0%)
Adjuvant	18 (56.3%)
Hormone therapy	9 (28.1%)
Neoadjuvant	0 (0.0%)
Adjuvant	9 (28.1)
Trastuzumab	32 (100%)
Median time from the prior last dose of trastuzumab to enrollment, months (range) Median cycles of prior trastuzumab treatment (range)	21.4 (6.4-95.9) 18.0 (3.0-63.0)
Previous surgery for primary breast cancer, n (%)	
Mastectomy	26 (81.3%)
Lumpectomy	8 (25.0%)
Other	2* (6.3%)
Type of recurrence, n (%)	
Local	2 (6.3%)
Regional	6 (18.8%)
Distant	28 (87.5%)
Lung	11 (34.4%)
Liver	9 (28.1%)
Bone	9 (28.1%)
Median LVEF, % (range)	66 (59-73)

Among the enrolled 32 patients, 28 patients finished the study according to the protocol. Four patients did not finish the study per protocol for reasons of refusing to continue treatment/withdrawing the informed consent form (2 patients), not returning for subsequent treatment (1 patient), or having previous accumulative dose of anthracyclines higher than the range allowed in inclusion criteria (1 patient). During this study, the median treatment duration with trastuzumab in combination with paclitaxel or docetaxel was 33.5 weeks ranging from 1 week to 148 weeks among which 22 patients received paclitaxel for 1-33 weeks, and 10 patients received docetaxel for 3-30 weeks.

### Primary endpoint results

**Figure 1:**
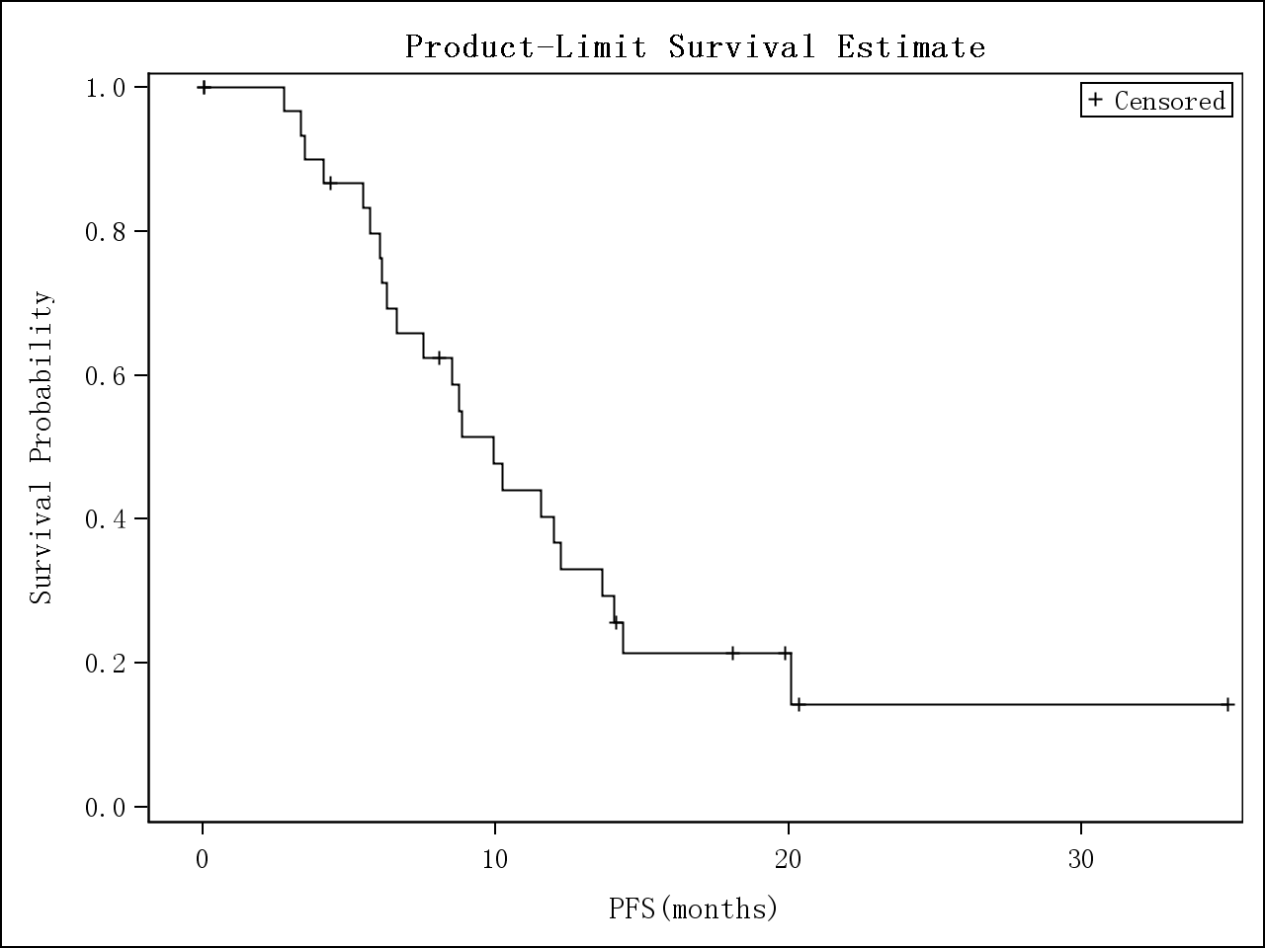
PFS Survival Curve (ITT) Kaplan-Meier curve of progression-free survival is shown.

As shown in Figure 1, the median PFS was 9.9 months (95% CI, 6.28 −13.63 months). Among all the 32 enrolled ITT patients, 23 patients (71.9%) had PFS endpoint events including 5 deaths (21.7%, 5/23) following progression of disease (PD). The remaining 9 patients (28.1%) had not reached the PFS endpoint events so their PFS was treated as censored data.

### Secondary endpoint results

**Figure 2:**
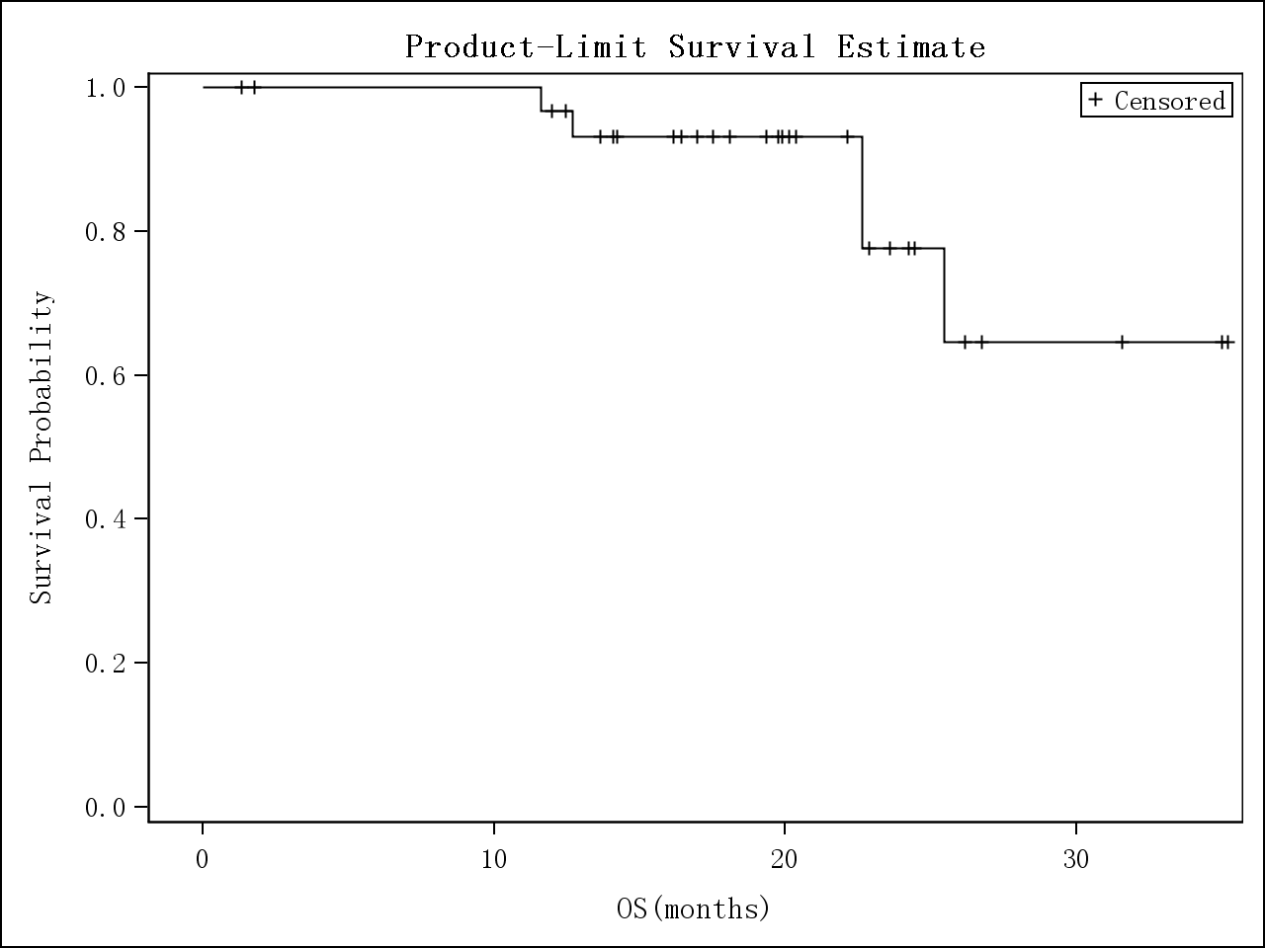
Overall Survival Curves (ITT) Kaplan-Meier curve of overall survival is shown

In ITT population of the 32 patients, the overall response rate (ORR) (calculated as complete response [CR]+partial response [PR]) was 81.3 % (26/32), 95% CI (63.6% - 92.8%) and clinical benefit rate (CBR) (calculated as CR+PR+stable disease [SD] ≥ 6 months) was 81.3% (26/32), 95% CI (63.6% - 92.8%). Duration of response (DOR) and OS were analyzed for the studied 32 patients, among which 26 patients (81.25%) had a treatment response (ITT = 26) (Table 2). During the DOR of these 26 patients, progression of disease after response was observed in 19 patients (73.1%) while PD or death was not observed in the remaining 7 patients (26.9%). The median DOR was 9.8 months with 95% CI (5.82 - 11.60 months). The median time to progression (TTP) was 9.9 months with 95% CI (6.28 - 13.63 months) (Table 2). Among ITT, death was observed in 5 patients (15.6%), and for the remaining patients, follow-up time of last survival was used as the truncation. Though median OS was not obtained, the 25% OS time was 25.5 months and the median OS follow-up time was 20.1 months (Table 2). The OS survival curve is shown in Figure 2. We performed exploratory analyses of progression-free survival across hormone receptor status, including estrogen receptor (ER) and progesterone receptor (PR). There were 24 patients with ER-/PR- and the median PFS was 8.74 months, 95% CI (6.05 - 11.53 months), 8 patients with ER+/PR+ and the median PFS was 14.03 months, 95% CI (3.35, -) respectively.

**Table 2:** Secondary endpoint results

		
		Result	95%CI
**ORR (ITT, N=32)**	**CR+PR, n (%)**	**26 (81.3%)**	**63.6%, 92.8%**
	CR, n (%)	3 (9.4%)	2.0%, 25.0%
	PR, n (%)	23 (71.9%)	53.3%, 86.3%
	SD, n (%)	4 (12.5%)	3.5%, 29.0%
	PD, n (%)	0	
	Unknown, n (%)	2 (6.3%)	0.8%, 20.8%
**CBR (ITT, N=32)**	**CR+PR+SD≥6 months**	**26 (81.3%)**	**63.6%, 92.8%**
**DOR (ITT, N=26)**	**Median (months)**	**9.8**	**5.82,11.60**
	Event number	19 (73.1%)	
	Censored number	7 (26.9%)	
	*No PD, no death*	7 (100.0%)	
**TTP (ITT, N=32)**	**Median (months)**	**9.9**	**6.28,13.63**
**OS (ITT, N=32)**	**Median (months)**	**NA**	**22.64, NA**
	Mean (months)	24.1 (0.75)	
	OS Event Number	5 (15.6%)	
	OS Censored Number	27 (84.4%)	
	*No death*	27 (100.0%)	
	Median follow-up time (Months)	20.1	

### Safety assessments

Overall, the safety profile of trastuzumab re-treatment in combination with a taxane as was acceptable and no new safety warning signal of trastuzumab was observed in this study. No significant changes after treatment compared to baseline was found for ECOG score, vital signs or body weight, and nor congestive heart failure was observed in any case in the study.

In the safety set (SS) population of 32 patients, there were a total of 308 cases of adverse event (AE) observed, and 30 subjects experienced at least once AE, resulting in an incidence rate 93.8% (Table 3). According to National Cancer Institute Common Toxicity Criteria Adverse Events version 3.0 (NCI CTC AE v3.0) grading, the portion with severity of grade III and above among total AEs was 22.1% (68/308). The common AEs at any grade with at least 20% occurrence include leukopenia (*n* = 19, 59.4%), neutropenia (*n* = 18, 56.3%), hypoaesthesia (*n* = 11, 34.4%), granulocytopenia (*n* = 10, 31.3%), asthenia (*n* = 7, 21.9%), and alopecia (*n* = 7, 21.9%). A detailed list of AEs by their severity is shown in Table 3. There were 6 cases of serious adverse event (SAE) observed in 5 patients, including infection (grade III), upper respiratory infection (grade III), leukopenia (grade IV), neutropenia (grade IV), cataract (grade III), and suicide (grade V), respectively.

**Table 3:** AE and SAE Summary (SS)

	SS (*N*=32)		
Type of adverse events	Patients with at least one event, n (%)	Severity by Grade	Number of events
			
**Any AE**	30 (93.8%)	I, II, III, IV, V	308
**Adverse drug reaction**	26 (81.3%)		239
**AE leading to drug withdrawal**	1 (3.1%)		1
**AE leading to death**	1 (3.1%)		1
**SAE**	5 (15.6%)		6
* Infection*	1 (3.1%)	III	1
* Upper respiratory tract infection*	1 (3.1%)	III	1
* Completed suicide*	1 (3.1%)	V	1
* Leukopenia*	1 (3.1%)	IV	1
* Neutropenia*	1 (3.1%)	IV	1
* Cataract*	1 (3.1%)	III	1
**AEs in at least 10% patients, by System Organ Class (SOC)**			
** Investigations**			
Alanine aminotransferase increased	4 (12.5%)	I, II	
** Nervous System Disorders**			
Hypoaesthesia	11 (34.4%)	I, II, III	
** Injury, Poisoning and Procedural Complications**			
Neurotoxicity	4 (12.5%)	I, II, III	
** Respiratory, Thoracic and Mediastinal Disorders**			
Cough	4 (12.5%)	I, II	
** Skin and Subcutaneous Tissue Disorders**			
Alopecia	7 (21.9%)	I, II	
Nail disorder	5 (15.6%)	I, II	
** General Disorders and Administration Site Reactions**			
Fever	6 (18.8%)	I, II	
Asthenia	7 (21.9%)	I, II, III	
Oedema peripheral	5 (15.6%)	I, II	
** Gastrointestinal Disorders**			
Diarrhea	5 (15.6%)	I, II	
Vomiting	4 (12.5%)	I, II	
Hypophagia	5 (15.6%)	I, II	
** Blood and Lymphatic System Disorders**			
Leukopenia	19 (59.4%)	II, III, IV	
Granulocytopenia	10 (31.3%)	II, III, IV	
Neutropenia	18 (56.3%)	II, III, IV	

The cases of adverse drug reaction (ADR) were also assessed (Table 3). There were a total of 239 ADR occurrences, contributing to an incidence rate of displaying at least one ADR of 81.3% (26/32). According to NCI CTC AE v3.0 severity scale, the incidence rate of grade III ADRs or above was 20.9% (50/239), which included leukopenia (*n* = 15, 46.9%), neutropenia (*n* = 16, 50%), granulocytopenia (*n* = 10, 31.3%), and fatigue (*n* = 2, 6.2%).

There was one drug withdrawal due to adverse event (3.2%), in which the subject withdrew drug treatment due to grade II headache. There were 5 death cases (15.62%) due to PD totally. In SS, asymptomatic Left Ventricular Ejection Fraction (LVEF) decrease of 10-18% compared to baseline was observed in 8 patients (25.0%), and any case with LVEF less than 40% was not found in this study.

## DISCUSSION

This prospective single-arm, multicenter trial demonstrated the efficacy and safety of re-treatment with first-line trastuzumab in 32 HER2-positive Chinese patients who had prior trastuzumab-based therapy in (neo)adjuvant settings.

Our efficacy results of trastuzumab re-treatment are comparable to previously reported data from studies involving patients no matter of being trastuzumab naive or of being pre-exposed. In the study H0648g, trastuzumab plus chemotherapy treatment resulted in TTP of 7.4 months, ORR of 50%, DOR of 9.1 months and OS of 25.1 months [10]. In the phase II randomized HERTAX Trial [20], the standard treatment of trastuzumab in combination with docetaxel resulted in a median PFS of 9.4 months and ORR of 79%, similar to our PFS of 9.9 months and ORR of 81.3%. Additional trials including Trial M77001 (showing ORR 61%, OS 31.2 months, TTP 11.7 months, DOR 11.7 months) [11] and other clinical studies investigating trastuzumab adjuvant or monotherapy [8, 21, 22] support the key importance of trastuzumab in treating HER2-positive early-stage and metastatic breast cancer. In these prospective trials, the included patients had been trastuzumab naïve. In present study, the enrolled patients had relapsed from trastuzumab-based (neo)adjuvant therapy, and yet still responded with median PFS and DOR of 9.9 and 9.8 months, respectively. This clearly supports the efficacy and importance of trastuzumab re-treatment in relapsed patients.

For re-treatment with first-line trastuzumab in patients previously treated trastuzumab, two retrospective [17, 18] and one prospective studies [19] have all shown effectiveness. The prospective study with trastuzumab in combination with docetaxel or paclitaxel by Lang et al showed a median PFS of 8.0 months, OS of 25.0 months in 41 patients after a 40-month median follow-up time [19], similar to our results. In the Italian multicenter retrospective cohort study of HER2-positive mBC, trastuzumab re-treatment of patients relapsing from previous (neo)adjuvant trastuzumab therapy showed a PFS of 12.0, ORR of 61.3%, CBR of 72.5%, and OS of 48.2 months [17]. In the retrospective study, trastuzumab as first-line therapy for HER2-positive mBC in patients previously treated with adjuvant trastuzumab showed a two-year OS of 60.0% [18]. Thus, these studies along with our data strongly support the notion that the tumor cells of the HER2-positive overexpressed metastatic breast cancer patients who relapsed after (neo)adjuvant trastuzumab treatment may still be sensitive to trastuzumab and that trastuzumab re-treatment is an important therapy for these patients.

Lapatinib in combination with capecitabine showed efficacy in women with HER2-positive, locally advanced or metastatic breast cancer that had progressed after initial trastuzumab-based therapy [23]. In a later single-arm, open-label trial EGF109491, lapatinib in combination with capecitabine only achieved a CBR of 57.7% and PFS of 6.34 months in 52 Chinese patients [24]. T-DM1 has been studied in various phase II [25, 26] and phase III [16] clinical trials showing its safety and efficacy in HER2-positive mBC patients with prior treatment with first-line trastuzumab adjuvant therapy. In the phase III EMILIA study of 991 HER2-positve mBC patients previously treated with adjuvant trastuzumab and taxane, T-DM1 treatment comparing with lapatinib/capecitabine combination therapy resulted in superior efficacy of median PFS of 9.6 months (*versus* 6.4 months) and a median OS of 30.9 months (*versus* 25.1 months) [16], with PFS being similar to our results for trastuzumab. Thus, compared to lapatinib in combination with capecitabine, trastuzumab plus chemotherapy as in our study would also have more favorable survival benefits in trastuzumab pre-treated HER2-positive mBC patients. A recently reported LUX-Breast1 study showed that trastuzumab-based therapy remains best choice for HER2-positive mBC patients who had progressed on or following adjuvant or first-line of trastuzumab compared with afatinib plus vinorelbine [27]. Pertuzumab, on the other hand, has been shown to be a complementary therapy to trastuzumab, as in the CLEOPATRA study [28]. Pertuzumab plus trastuzumab plus docetaxel significantly prolonged survival as compared to placebo plus trastuzumab plus docetaxel treatment in HER2-positive mBC patients, with median PFS of 18.5 months and 12.4 months, respectively [28]. However, despite that T-DM1 and pertuzumab may serve as effective alternatives to trastuzumab in HER2-positive patients, neither is currently available in China.

The safety assessments in our study show that trastuzumab re-treatment in combination with a taxane was safe in general as no new safety warning signal of trastuzumab was observed. Though 30 subjects experienced at least once AE, most of them belonged to blood and lymphatic system disorders including leukopenia, neutropenia and granulocytopenia, similar to previous reports [10, 11]. Our results indicate only a mild asymptomatic LVEF decrease of 10-18% compared to baseline in 8 patients. It is possible that the low cardiac event rate in our study was also due to the inclusion criteria of LVEF > 50%, as trastuzumab re-treatment study [19] and the CLEOPATRA trial [28, 29] as well as other trastuzumab studies. This is consistent with recent reviews and studies suggesting that the cardiac risk of trastuzumab treatment is low [30, 31]. Lapatinib in combination with capecitabine, however, was reported to have fatal AEs as well as AEs (13%) leading to discontinuation of treatment [23]. Lapatinib with a taxane was also found to be associated with more grade III/IV diarrhea and rash adverse events (AEs) than trastuzumab/taxane combination [32]. Our study also provided valuable ethnic safety data for trastuzumab re-treatment. Asian women may have a lower activity of CYP3A4, the P450 enzyme associated with docetaxel metabolism, compared with Caucasian women [33], possibly contributing to inter-ethnic differences in docetaxel clearance and toxicity [34]. However, our results demonstrated that re-treatment of trastuzumab in combination with a taxane at standard doses was well tolerated in Chinese patients.

Relapse of mBC that had responded to trastuzumab seems to involve complex mechanisms. Factors contributing to HER2 resistance [35-38] may be ruled out, since relapsed patients would be refractory to trastuzumab re-treatment. Cessation of trastuzumab therapy may remove its anti-angiogenic activity, leading to upregulation of vascular endothelial growth factor and other angiogenic factors [39]. Withdrawal of trastuzumab treatment may also diminish its stimulatory effect on effector cells leading to decreased antibody-dependent cell-mediated cytotoxicity (ADCC) and increased tumor growth [40]. Therefore, the effectiveness of trastuzumab re-treatment in relapsed patients in the present and previous studies warrants further *in vitro* and *in vivo* mechanistic investigations.

It should be noted that our study was limited by its single arm, non-comparativeness, and the enrolled patient size being relatively small. Though the efficacy data were similar to the study by Lang et al [19], our patient enrollment was restricted to Chinese population. In addition, for the subgroup analysis across hormone receptor status, HR+ population showed longer median PFS compared with HR- population, but there were only 8 HR+ patients *vs* 24 HR- patients, thus we would not conclude that HER2+HR+ patients benefit more from first-line trastuzumab re-treatment in this study. Moreover, the overall survival data could not be obtained in our study. Longer follow up time may also be needed for OS determination, given that long-term [41] and continuous treatment [42] outcomes of first-line trastuzumab-containing therapy in HER2-positive metastatic breast cancer patients have been reported as efficacious and safe. Thus additional clinical investigations of larger size and longer follow-up time may be conducted to fully assess the benefit of this trastuzumab re-treatment regimen.

In summary, re-treatment with first-line trastuzumab in combination with a taxane is an effective regimen for patients with HER2 positive metastatic breast cancer relapsed after (neo)adjuvant trastuzumab. The safety profile of trastuzumab re-treatment was good and the adverse reactions were tolerable and manageable. The data confirmed the importance and significance of continued trastuzumab-based anti-HER2 therapy in HER2-positve breast cancer.

## MATERIALS AND METHODS

### Study design

This present study is a prospective, open label, multicenter, single arm trial to investigate the clinical outcomes of re-treatment with first-line trastuzumab in combination with a taxane in HER2 positive mBC patients who had relapsed after receiving trastuzumab in the (neo)adjuvant setting for HER2-positive eBC. It is registered with ClinicalTrials.gov (NCT01301729). This study was conducted in accordance with Good Clinical Practice and the Declaration of Helsinki. The protocol was approved by Institutional Ethics Committee/Institutional Review Board of each participating study site. All patients provided written informed consent.

The primary endpoint was PFS, defined as the time from registration of the enrollment to time of first documented disease progression or death, whichever occurs first. The secondary efficacy endpoints included: ORR (defined as the percentage of patients with a either CR or PR according to the Response Evaluation Criteria in Solid Tumors [RECIST] 1.0), CBR (defined as CR + PR + SD for at least 6 months), DOR (defined as the time from when the response [CR or PR] was first noted until date of documented progression or death), TTP (defined as the number of days from date of registration of enrollment to date of disease progression) and OS (defined as the number of days from date of registration of the enrollment to date of death from any cause).

### Patient eligibility

#### Inclusion criteria

Female patients of at least 18 years old to be included in the study must meet at least the following criteria: diagnosed with recurrent local or metastatic breast cancer; having HER2 positive primary disease(central lab retesting as 3+ HER2 overexpression measured by immunohistochemistry, or HER2 amplification measured by FISH); had completed at least 9 weeks of trastuzumab treatment for HER2 positive eBC in adjuvant or neoadjuvant setting; relapsed ≥6 months after discontinuation of last trastuzumab and/or chemotherapy in the (neo)adjuvant setting for HER2 positive eBC; measurable disease according to RECIST criteria 1.0; ECOG performance status of 0-2; life expectancy ≥ 12 weeks; maximum cumulative dose of doxorubicin ≤360 mg/m2 or maximum cumulative dose of epirubicin ≤720 mg/m2 or no prior anthracyclines (due to known resistance to prior anthracyclines or toxicity); baseline LVEF ≥ 50% or within normal limits (WNL) at a given institution; no prior history of myocardial infarction within 12 months, or unstable angina pectoris, or cardiac insufficiency (New York Heart Association Class III - IV), or uncontrolled arrhythmia at time of inclusion (Patients with symptomatic congestive heart failure may be included at the investigator's discretion if occurrence was > 6 months prior to enrollment). If there was previous surgery or radiotherapy, patients must have subsequently waited at least 3 weeks. In addition, patients must sign written informed consent obtained prior to any study specific procedures and be able to comply with the protocol.

#### Exclusion criteria

Patients with any of the following were excluded from the study: in pregnancy or lactation; had prior chemotherapy for metastatic breast cancer (Prior surgery, endocrine therapy and/or radiotherapy were allowed); pleural effusions, ascites, or bone lesions as the only manifestation of disease; brain metastatic disease; invasive malignancy (including second primary breast cancer) other than metastatic breast cancer which could interfere with compliance with the protocol or interpretation of results; inadequate bone marrow, hepatic and renal functions; had prior treatment with anti-HER therapies other than (neo)adjuvant trastuzumab; had treatment with any investigational drug within 30 days before the beginning of treatment with study drug; with known allergy or severe reactions to trastuzumab or its constituents; history of uncontrolled seizures, central nervous system disorders or psychiatric disability, which could hinder ability to give informed consent, or compliance with study therapy. Further, patients with serious illness or medical conditions were excluded, including history or current congestive heart failure (New York Heart Association Class III-IV), unstable angina pectoris, myocardial infarction in the last 12 months, poorly controlled hypertension (systolic > 180 mmHg or diastolic > 100 mmHg), clinically significant valvular heart disease, or high-risk uncontrolled arrhythmias, active serious uncontrolled infections, poorly controlled diabetes mellitus, active peptic ulcer or other contraindication to high dose of corticosteroid therapy such as herpes zoster, cirrhosis.

### Treatment regimen

Treatment and monitoring were recorded by intervals of periods, in that each period lasted 3 weeks. Trastuzumab was administered once weekly, starting at 4 mg/kg loading dose on day 1 by intravenous infusion over 90 min and followed by subsequent 2 mg/kg intravenous infusion over 30 min as maintenance doses, given once weekly starting on day 8, until disease progression. After initial dose of trastuzumab, the patient was observed for 6 hours. If adverse event did not occur after the initial infusion, the subsequent observation period of administration was shortened to 2 hours after infusion. Body weight was measured before each dose of trastuzumab and it was used for actual dose calculation.

The choice of the taxane (docetaxel and paclitaxel) to be used in combination with trastuzumab was made by the investigator's clinical judgement for each patient. Paclitaxel and docetaxel treatment may begin within 24 hours after administration of trastuzumab or on the same day (at least 30 min after trastuzumab infusion). Subsequently administration may be repeated every 21 days up to 6 times as judged by the investigator. Docetaxel (Taxotere) was intravenously infused for more than 1 hour with the initial dose of 100 mg/m2 (±10%), q3w. Paclitaxel (Taxol) was intravenously infused for more than 3 hours with the initial dose of 90 mg/m2 (±10%), q1w. Taxane was first administered for 6 periods (18 weeks) until unacceptable toxicities or the patient's withdrawal from study.

### Assessments

Tumor response was evaluated according to RECIST 1.0, anti-tumor efficacy were analyzed approximately 12 months after last enrolled patient. Patients were assessed for disease response or progression every 6 weeks (2 cycles) until disease progression or death being documented. Objective responses were confirmed with a repeat examination within 4 weeks. Survival data were collected after treatment completion or after the progression of disease, and was done once every year and one year after the enrollment of the last patient.

Adverse events were continuously monitored, recorded and graded according to the NCI CTC AE v3.0, and for the case of congestive heart failure, according to New York Heart Association (NYHA) classification. Safety profile including changes in LVEF and incidence of symptomatic heart failure of trastuzumab with a taxane in this patient population was determined.

### Statistical analysis

Based on the time to disease progression for patients treated with trastuzumab plus taxane being 7.1 months in the H0648g study and 10.6 months in the M77001 study [10, 11], a PFS of 8-10 months was considered to be acceptable. With a desired PFS of 9 months in our study, a sample size of 50 patients was believed to be sufficient to estimate the PFS with 95% CI of 6.9 months-12.1 months. Analysis population was based on 2 analysis sets: ITT and Safety Set (SS). ITT population was defined as all the patients who were eligible through screening, register and enter the trial, which was used for efficacy analysis. Safety population is defined as all patients registered in the study and have taken at least one study drug, which was used for safety analysis. Statistical analysis was performed using SAS 9.2. Time-dependent endpoint analyses including PFS, TTP, OS and DOR was conducted by the Kaplan-Meier estimation. Median OS follow-up time was calculated by exchanging the event of OS occurrence with censored patients, after which the median time was estimated by Kaplan-Meier survival curve analysis. Objective response rates including ORR and CBR were presented along with 95% confidence interval. Patients without an event were censored at the last tumor assessment or last follow-up for disease progression at which they were known to be progression free. Patients without any post-baseline tumor assessment were censored at registration. Adverse events and baseline characteristic data were analyzed with descriptive statistics.
